# Notes and News

**Published:** 1898-02-12

**Authors:** 


### Notes and News.

(Collected and Communicated.)

### HOSPITAL MEETINGS.

London Fever Hospital.—Annual meeting of Governors, Friday, February 11th, 5 p m., at Hotel Victoria, Northumberland Avenue.

Seamen's Hospital.—The Annual Court of Governors will be held on Friday, February 25th, at 3 o'clock, at the Royal United Service Institution, Whitehall.

Royal Westminster Ophthalmic Hospital.—A dinner in aid of the Building Fund of this hospital is to be held at the Whitehall Rooms, Hotel Metropole, on April 30th, at which the Right Hon. W. Court Gully, Q.C., will preside.

The Stock Exchange fund in aid of the London Hospital is progressing well, the first list including over 60 subscribers of £52 10s. each.

The Duchess of Devonshire, as Mayoress of Eastbourne, accompanied by the Countess of Gosford, recently paid a visit to the Princess Alice Hospital.

The Duke of Fife has promised to preside on May 16th at the anniversary festival of the Orphan Working School to be held at the Hotel Metropole.

The North-West London Hospital suffered so seriously from decreased subscriptions last year that the treasurer, Mr. George Herring, has generously met the deficiency by a donation of £5,000.

Lord Knutsford distributed 500 Jubilee medals to members of the St. John Ambulance Corps, at Clerkenwell, on the 29th ult., in recognition of the good work done by them on Jubilee Day in the metropolis.

William Harding, M D.Edin., M.R.C.P., has been appointed medical superintendent of the Northampton County Asylum, Berrywood, in the place of Richard Green, F.R.C.P.Edin., who has resigned.

A new wing has been added to the Bishop Stortford Hospital in commemoration of the Diamond Jubilee. The four wards already existing have proved insufficient to accommodate the large number of cases applying for admission.

The foundation-stone of the new Isolation Hospital for Chester has just been laid by the Mayor. The building is on the pavilion plan, and is to be lighted by electricity, will accommodate 48 patients, and is to cost £14,400.

The baths at Weissenberg, in the Bernese Oberland, have been burnt down. Weissenberg is a well-known health resort for consumptives.

Madame Chassegros, known in Paris for many years as a protector of animals, has left the whole of her fortune—nearly three million francs—to the Society for Prevention of Cruelty to Animals.

Influenza, accompanied with pulmonary complications, is prevalent throughout Devon and Cornwall. Truro, Falmouth, and Devonport are amongst the towns most seriously affected, and at the latter place many cases have occurred among the boys on Her Majesty's training-ships.

The wife of Bishop Walsh is appealing for funds to establish a hospital for women in Mauritius, She says the suffering and mortality amongst the sugar workers of the island is appalling. She proposes that a hospital, a dispensary, and a qualified medical woman should be provided, and asks for £1,000 toward this object.

A smart outbreak of small-pox has occurred at Middlesbrough, where 150 cases have already been reported. Cases have also occurred at Thornaby and Ormesby. The disease is said to have been introduced into the district by a travelling show. The Middlesbrough Isolation Hospital has been cleared, and is to be devoted to small-pox cases, and four additional corrugated iron pavilions are being erected.

It has been decided by the court of directors, after very careful consideration, to close the Royal Sea-Bathing Infirmary as early in March next as is possible, for the purpose of carrying out certain necessary structural alterations and repairs in the old buildings and in the general sanitary arrangements of the hospital. It is hoped that the hospital may be re-opened in June or July next, due notice of which will be given.

General Sir Henry Norman delivered the prizes to the successful candidates for the Army Medical Staff at Netley. The following gentlemen passed the examination, and are placed in order of merit: H. O. B. Browne-Mason, who gained the Maclean prize for clinical and ward work; F. S. Penny, who gained the pathology and Parke's memorial bronze medal; B. Watts, H. G. Martin, J. G. Berne, F. F. Carroll, J. D. G. Macpherson, W. P. Gwynn, S. de C. O'Grady, A. H. O. Young, E. A. Bourke, M. M. Lowsley, A. C. Lupton, G. B. Carter, N. H. Ross, P. H. Collingwood, and C. J. O'Gorman. The following is a list of successful candidates for the Indian Medical Service: T. H. Delany, who gained the pathology prize and the Herbert prize; J. W. F. Rait, who gained the Montefiore medal and prize of 20 guineas, and the de Chaumont prize in hygiene; S. R. Douglas; E. J. O'Meara, who gained the Martin memorial gold medal; G. Tate, who gained the second Montefiore prize in surgery; R. F. Baird, A. T. Gage, G. C. Laing, G. MacPherson, S. Hunt, A. G. Sargent, W. H. Cox, de V. Condon, H. A. J. Gidney, H. Kirkpatrick, F. D. S. Fayrer, P. H. Chitale, and W. Lethbridge.

